# Patients with PWS and related syndromes display differentially methylated regions involved in neurodevelopmental and nutritional trajectory

**DOI:** 10.1186/s13148-021-01143-0

**Published:** 2021-08-13

**Authors:** Juliette Salles, Sanaa Eddiry, Emmanuelle Lacassagne, Virginie Laurier, Catherine Molinas, Éric Bieth, Nicolas Franchitto, Jean-Pierre Salles, Maithé Tauber

**Affiliations:** 1 Université de Toulouse, Toulouse, France; 2 Service de Psychiatrie et Psychologie, CHU de Toulouse, Toulouse, France; 3 Infinity (Toulouse Institute for Infectious and Inflammatory Diseases), INSERM UMR1291, CNRS UMR5051 , Université Paul Sabatier, Toulouse III, France; 4 Centre de Référence Prader-Willi, Hôpital Marin, APHP, Hendaye, France; 5 Centre de Référence du Syndrome de Prader-Willi et Syndromes avec Troubles du Comportement Alimentaire, Unité D’endocrinologie, Obésités, Maladies Osseuses, Génétique et Gynécologie Médicale, Hôpital des Enfants, CHU Toulouse, Toulouse, France; 6 Service de Génétique Médicale, Hôpital Purpan, CHU, 31059 Toulouse, France; 7 Service d’Addictologie Clinique, Urgences Réanimation Médecine, CHU de Toulouse, Toulouse, France; 8 Institut des Handicaps Neurologiques, Psychiatriques et Sensoriels, CHU de Toulouse, Toulouse, France

**Keywords:** Neurodevelopmental disorder, Genome-wide methylation analysis, Prader–Willi, *SNORD116*, *MAGEL2*

## Abstract

**Background:**

Prader–Willi syndrome is a rare genetic neurodevelopmental disorder caused by a paternal deficiency of maternally imprinted gene expression located in the chromosome 15q11–q13 region. Previous studies have demonstrated that several classes of neurodevelopmental disorders can be attributed to either over- or under-expression of specific genes that may lead to impairments in neuronal generation, differentiation, maturation and growth. Epigenetic changes that modify gene expression have been highlighted in these disorders. One recent study focused on epigenetic analysis and compared patients with PWS with patients with other imprinting disorders. No study, however, has yet focused on epigenetics in patients with PWS specifically by comparing the mutations associated with this syndrome.

**Objective:**

This study investigated the epigenetic modifications in patients with PWS and patients with PWS-related disorders caused by inactivation of two genes of the PWS chromosomal region*, SNORD116* and *MAGEL2*. Our approach also aimed to compare the epigenetic modifications in PWS and PWS-related disorders.

**Methods:**

We compared genome-wide methylation analysis (GWAS) in seven blood samples from patients with PWS phenotype (five with deletions of the PWS locus, one with a microdeletion of *SNORD116* and one with a frameshift mutation of *MAGEL2* presenting with Schaaf–Yang syndrome), as well as two control patients. Controls were infants that had been studied for suspicion of genetic diseases that was not confirmed by the genetic analysis and the clinical follow-up.

**Results:**

The analysis identified 29,234 differentially methylated cytosines, corresponding to 5,308 differentially methylated regions (DMRs), which matched with 2,280 genes. The DMRs in patients with PWS were associated with neurodevelopmental pathways, endocrine dysfunction and social and addictive processes consistent with the key features of the PWS phenotype. In addition, the separate analysis for the *SNORD116* and *MAGEL2* deletions revealed that the DMRs associated with the *SNORD116* microdeletion were found in genes implicated in metabolic pathways and nervous system development, whereas *MAGEL2* mutations mostly concerned genes involved in macromolecule biosynthesis.

**Conclusion:**

The PWS is associated with epigenetic modifications with differences in *SNORD116* and *MAGEL2* mutations, which seem to be relevant to the different associated phenotypes.

## Background

Prader–Willi syndrome (PWS) is a rare genetic neurodevelopmental disorder (NDD) caused by a paternal deficiency of maternally imprinted gene expression in the chromosome 15q11–q13 region [1]. This lack of gene expression occurs because of deletions of paternally inherited 15q11–q13 chromosomal region or because of the occurrence of maternal uniparental disomy (UPD). The 15q11.2–q13 region can be divided into distinct regions that are delineated by three common deletion breakpoints (BP), a proximal non-imprinted region between the two common proximal breakpoints (BP1 and BP2) containing four biparentally expressed genes, NIPA1, NIPA2, CYF1P1 and GCP5.94 and the “PWS paternal-only expressed region” between BP2 and BP3 containing five polypeptide coding genes (MKRN3, MAGEL2, NECDIN and the bicistronic SNURF-SNRPN); C15orf2; a cluster of C/D box small nucleolar RNA genes (snoRNAs); and several antisense transcripts (including the antisense transcript to UBE3A). These three breakpoints cause two classes of deletions commonly called type 1 or long deletion when they extend from BP1 to BP3 and type 2 or short deletion when they extend between BP2 and BP3. In childhood and adulthood, patients with PWS present severe obesity related to eating disorders and obsession for food, endocrine dysfunction (impaired sexual development and growth, central hypothyroidism, rare adrenal insufficiency) and intellectual disabilities. It is now acknowledged that an impaired development and function of the hypothalamus may explain the specific features of the PWS phenotype (art lancet endocrinol metab M Tauber 2021).

The PWS phenotype occurs with a specific nutritional trajectory from anorexia at birth to hyperphagia in children and adults. The literature on PWS is broad concerning the cognitive impairments, social deficits [2] and brain metabolism modifications [3] that occur during neurodevelopment, especially those involving hypothalamic dysfunction with structural abnormalities [4] and impaired hypothalamic brain connectivity [5]. In addition, this syndrome is associated with several psychiatric dimensions [6] that can be connected to NDDs such as the autism spectrum disorders (ASD) [7] and attention deficit hyperactivity disorder (ADHD) [8]. The role of epigenetics in NDDs has been highlighted, and some authors have suggested that these disorders be classified as “epigenetic” as they are at the interface between genetics and environmental risk factors [9].

Several studies have indicated that imprinted genes play important roles in the postnatal processes that may be particularly responsive to environmental influences [10–12]. Genomic imprinting is a form of epigenetic inheritance whereby the regulation of the imprinting-associated differentially methylated regions (iDMRs) is dependent on the sex of the transmitting parent [13]. However, DMRs can be identified in loci other than iDMRs. Indeed, a genome-wide methylation analysis (GWMA) study in Silver–Russell syndrome (SRS) patients showed that DMRs were found at the IGF2/H19 locus and that 116 DMRs were located on other chromosomes [14]. Moreover, a GWMA in patients with various imprinting disorders identified patients with multilocus imprinting disturbances (MLID) [15]. Given the potentially shared epigenetic regulation in imprinting disorders, Hara‑Isono et al. recently investigated the methylation signatures associated with the overlapping phenotypes of three imprinting disorders in SRS, temple syndrome (TS14) and PWS. However, no methylation signatures were found to be shared by these three syndromes [16]. We found that these imprinting disorders shared phenotypical similarities concerning growth, development and endocrine and metabolism dysfunctions but showed differences in their neurodevelopmental trajectories. Adult patients with PWS present intellectual disability, social impairment and emotional lability, whereas adult patients with TS14 classically present normal intellectual development and can expect to attend university [17]. We hypothesized that these phenotypical differences would partly explain the results of the authors.

Moreover, although the phenotypical characteristics may differ between syndromes, they may also vary within the same syndrome. Patients with PWS and PWS-related disorders present complete or partial PWS phenotypes depending on the type of mutation in the 15q11–q13 region. For example, a patient described with a *SNORD116* microdeletion (MD) [18] displays a complete PWS phenotype and patients with *MAGEL2* mutations present with Schaaf–Yang syndrome (SYS), which comprises such PWS features as an early phase of poor feeding, endocrine dysfunction and more severe ASD features.

Considering these data, we aimed to (i) specify the methylation signature in PWS and PWS-related syndromes by considering a group with different mutations of the 15q11–q13 region including *SNORD116* MD and *MAGEL2* mutation, (ii) associate the signatures with biological pathways and clinical features and (iii) specify the methylation signatures with the two mutations of *SNORD116* MD and *MAGEL2*.

## Results

### Clinical features

The clinical features, including age and gender, and the genetic data of the patients are presented in Table 1. All patients displayed a complete or partial PWS phenotype. The PWS group included two infants, one child and four adults; two of the patients were female and five were male. The control group was composed of two infants, one female and one male.

**Table 1:** Clinical features of the patients; NA: non-applicable

Group	Age category	Gender	Genotype
PWS1	Infant (1 Year)	Male	Deletion type1
PWS2	Child (10 year)	Male	Deletion type1
PWS3	Adult (27 years)	Female	Deletion type2
PWS4	Adult (32 years)	Male	Deletion type2
PWS5	Infant (1 year)	Male	Uniparental disomy
PWS6	Adult (32 years)	Female	*SNORD116* microdeletion
PWS7	Adult (21 years)	Male	*MAGEL2* mutation
Control	Infant (1 year)	Male	NA
Control	Infant (1 year)	Female	NA

### Distribution of the DMRs

We performed an analysis of the DMRs by RRBS approach which compared all the patients with PWS with controls. The analysis tested 1,971,050 cytosines and identified 29,234 differentially methylated cytosines, corresponding to 5,308 DMRs. These DMRs matched with 2,280 genes. Table 2 describes the top 50 of the hypomethylated genes and the top 50 of the hypermethylated genes.

**Table 2:** Description of the top 100 of the gene with the higher methylation difference (50 hypermethylated genes and 50 hypomethylated genes)

	Genes	Methylation difference (%)	p value
Top 50 of the hypomethylated genes	GGT6	− 100	0.000514363
	GRK5	− 100	0.000,635,702
	KCNJ15	− 100	0.000,170,544
	LOC101927824	− 100	5.8E−23
	MAP1B	− 100	0.000,405,688
	PHRF1	− 100	0.000,635,702
	RAB3GAP1	− 100	0.000,621,357
	ZNF592	− 100	0.000,405,688
	ITGA9− AS1	− 97,44,493,917	0.0,000,013
	LYST	− 97,44,493,917	0.0,000,013
	APC2	− 97,3,698,163	0.00,109,708
	PSMD12	− 97,3,698,163	0.00,109,708
	LOC728743	− 96,97,178,877	0.000,564,938
	TTC39B	− 96,97,178,877	0.000,564,938
	CXADR	− 96,68,781,868	0.0,000,176
	TDRD10	− 96,00,217,622	0.0,000,356
	CCER2	− 95,55,845,589	0.00,000,562
	TBC1D22A	− 95,55,845,589	0.00,000,562
	ZNF510	− 92,27,324,274	0.000,437,373
	ABCG4	− 92,05,494,615	2.35883E−09
	GJC1	− 92,05,494,615	2.35883E−09
	CYP27A1	− 91,74,804,204	5.85E−08
	WDR78	− 91,36,703,468	0.000,000,003
	LOC102723665	− 90,51,175,406	0.000,568,276
	SH3D19	− 90,51,175,406	0.000,568,276
	ACOT9	− 89,67,481,584	2.12E−49
	LINC00476	− 89,67,481,584	2.12E−49
	CACFD1	− 87,25,081,098	0.00,000,197
	MTCP1	− 87,25,081,098	0.00,000,197
	GOLGA7B	− 87,13,880,766	2.60667E−05
	GSDMD	− 86,01,727,055	0.000,527,908
	HUS1	− 86,01,727,055	0.000,527,908
	UBXN6	− 85,98,645,224	0.000,102,859
	CERCAM	− 85,61,137,856	0.000,358,764
	DOCK11	− 85,61,137,856	0.000,358,764
	C3P1	− 84,44,523,505	1.52E−09
	TBCD	− 84,44,523,505	1.52E−09
	C1QTNF9	− 84,32,653,935	0.000,000,467
	IARS1	− 84,11,532,069	2.29667E−06
	BSPRY	− 83,73,678,607	0.000,156,053
	TFE3	− 83,73,678,607	0.000,156,053
	SMARCA2	− 83,57,182,955	0.000,004,115
	CCDC62	− 83,11,684,461	9.48193E−09
	FAM117B	− 83,11,684,461	9.48193E−09
	DENND2B	− 83,11,642,437	0.000,501,791
	ZNF462	− 82,62,363,647	0.000,132,237
	ARID2	− 81,88,091,076	0.000,330,603
	LRRTM1	− 81,88,091,076	0.000,330,603
	SPON1	− 80,65,841,688	0.00,000,314
	VPS4B	− 80,51,171,134	0.0,000,114
Top 50 of the genes hypomethylated	EFCAB2	77,73,232,951	0.001,120,107
	DGKD	78,91,166,751	0.000,352,854
	LOC283683	78,91,166,751	0.000,352,854
	DLX2	81,76,085,867	0.000,213,868
	CAMSAP3	84,37,216,271	0.000,563,998
	SLC25A10	84,37,216,271	0.000,563,998
	CACTIN-AS1	84,99,735,616	3.18E−08
	RPS7	85,01,990,768	0.0,004,312
	CCDC110	85,65,159,895	0.000,654,831
	TMEM184B	87,1,437,235	0.000,244,851
	CYBC1	88,63,637,014	0.000,616,386
	MUC16	88,63,637,014	0.000,616,386
	MLIP	88,69,043,414	0.000,146,656
	DOCK10	90,28,688,782	0.0,000,496
	MMP17	90,28,688,782	0.0,000,496
	HIF3A	90,90,923,198	0.0,000,676
	SMOX	90,90,923,198	0.0,000,676
	GMCL1	94,26,290,015	0.00,000,577
	ITPR2	94,26,290,015	0.00,000,577
	LINC00205	98,14,808,839	1.0035E−06
	SPON2	98,14,808,839	1.0035E−06
	TARBP2	98,27,592,303	0.000,171,639
	EMD	99,84,144,366	0.000,046
	BCOR	100	0.000,443,279
	CDC42BPA	100	0.0,000,146
	FAF1	100	0.000,133,428
	FGD5	100	0.0,000,146
	GALNT8	100	0.000,358,708
	GSEC	100	0.0,000,634
	HMGXB3	100	0.000,204,717
	INTS11	100	0.0,000,194
	KMT2A	100	0.000,063
	MAN2A1	100	0.0,000,981
	NFKBIL1	100	0.000,133,428
	POMT2	100	0.0,000,159
	PPM1B	100	0.000,358,708
	PTPRE	100	0.000,725,654
	RASA2	100	0.000,533,106
	SDC3	100	0.0,000,566
	SERPINB9P1	100	0.0,000,566
	SH2B2	100	1.12E−19
	SLC27A2	100	4.92E−21
	TMEM222	100	1.09E−26
	TMEM242	100	0.001,111,454
	TMUB2	100	0.000,063
	TNRC6A	100	0.000,533,106
	TTC23	100	0.0,000,981
	TTLL6	100	0.0,000,634
	UNC5A	100	0.0,000,159
	XPR1	100	0.001,111,454

The total distribution for the DMRs indicated that 34% were located in intergenic regions with the following repartition: 58% were located in transcription start sites (TSSs), which are regions that exert a great influence on gene expression regulation [19], and 42% were located in the transposable elements (TEs), which are the highly repetitive DNA sequences that constitute more than 50% of the human genome and contain about 52% of all CpG dinucleotides [20].

Sixty-six percent of the DMRs were located in intragenic regions, with a repartition of 47% in the exons and 53% in the introns. The following analysis in the intragenic regions was conducted in order to identify the associated biological pathways.

**Fig. 1:**
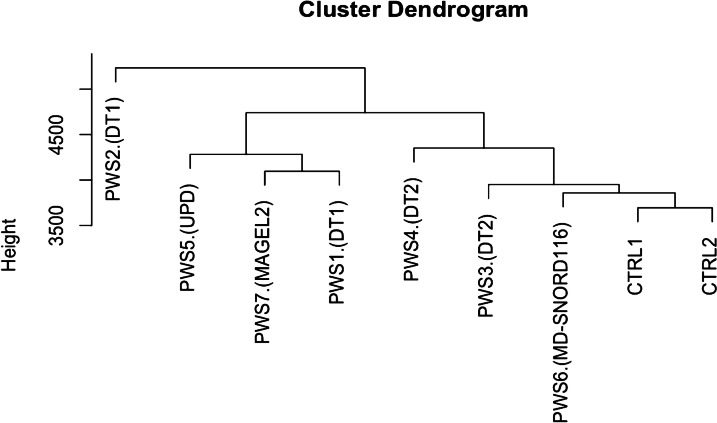
Results of hierarchical clustering including the gender, the age and the DMRs; the dendrogram includes, from right to left: CTRL2: female infant control, CRTL1: male infant control, PWS6 (SNORD116 MD): female adult patient with SNORD116 deletion, PWS3: female adult patient with type 2 deletion, PWS4: male adult patient with type 2 deletion, PWS4: male adult patient with type2 deletion, PWS1: male infant patient with type 1 deletion, PWS7 (MAGEL2): male adult patient with MAGEL2 mutation, PWS5: male infant patient with uniparental disomy (UPD), PWS2: male child patient with type 1 deletion

Hierarchical clustering analysis of the DMRs showed that the two controls grouped together. In this analysis, age did not play a notable role in the clustering. Moving from the control cluster, we found the patient with the *SNORD116* MD and the two PWS patients with type 2 deletion (short deletion or DT2). Less close were the patients with type 1 deletion (long deletion or DT1), the patient with *MAGEL2* mutation and a patient with uniparental disomy (UPD) (Fig. 1). One individual with DT1 (PWS2) was independently clustered.

### Functional analyses of genes associated with DMRs and association with neurodevelopmental and nutritional trajectories

**Fig. 2:**
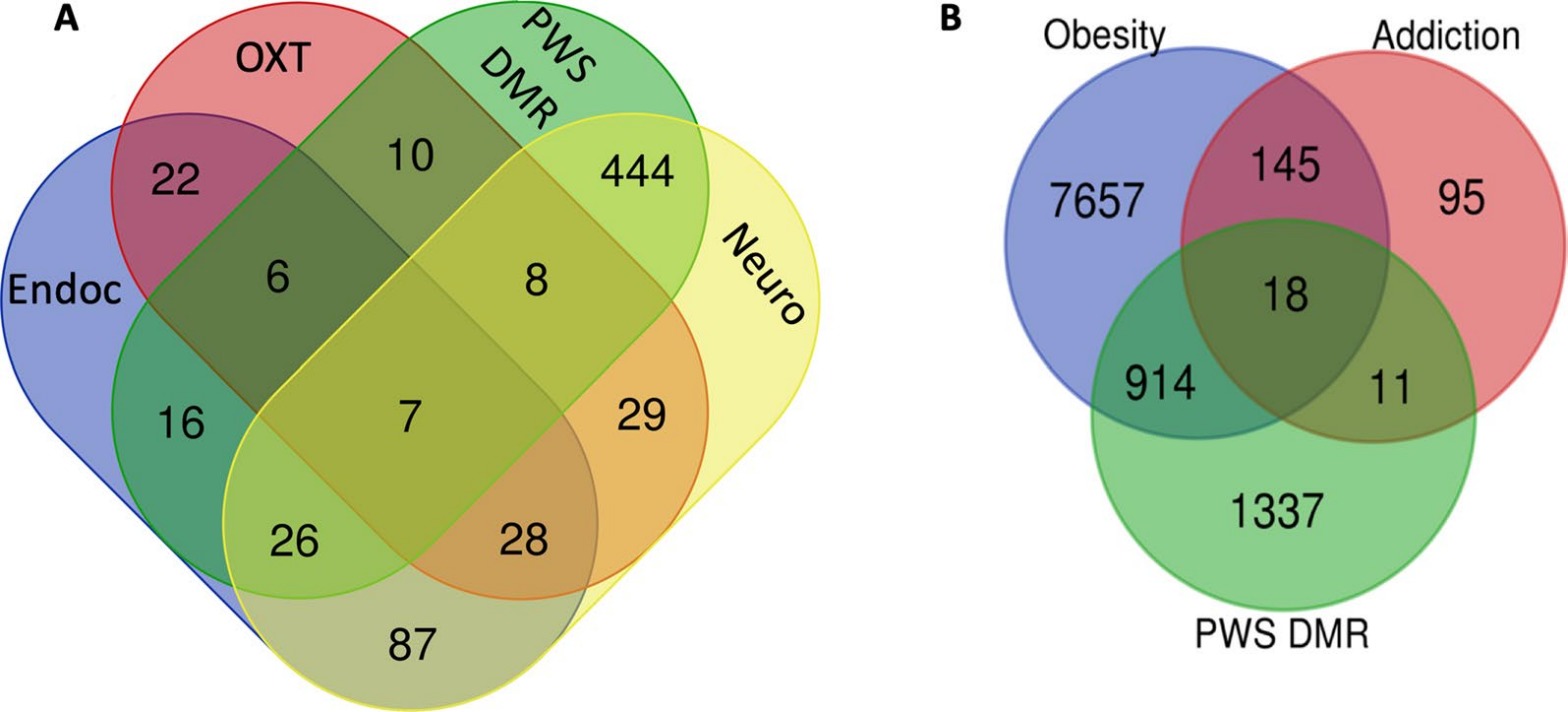
**a** Venn diagram for the PWS DMRs of the oxytocin pathway (OXT), nervous system development (Neuro), the endocrine pathway (Endoc) and the Prader–Willi differentially methylated regions (PWS DMRs). **b** Venn diagram of the genes implicated in addiction, obesity and the PWS DMRs

We performed a gene ontology functional pathways analysis that included the 2,280 genes corresponding to the DMRs. The most significant results for the functional pathways associated with the DMRs in PWS included biological processes and pathways related to nervous system development, generation of neurons and neurogenesis, anatomical structure development, synapses, aldosterone synthesis, Cushing syndrome, cortisol synthesis, cholinergic synapse, oxytocin signaling and endocrine resistance (Table 3). Four hundred and eighty-five (21%) of the differentially methylated genes corresponded to nervous system development. In addition, some genes involved in neurodevelopment overlapped with other systems related to the PWS phenotype (endocrine resistance and oxytocin pathway). Figure 2a illustrates this overlap.

**Table 3:** Top biological processes and KEEG pathway connected to the PWS DMRs

Biological process/KEGG pathway	GO/KEGG ID	Adjusted_p_value
Nervous system development	GO:0007399	1.23E−14
Generation of neurons	GO:0048699	1.30E−13
Neurogenesis	GO:0022008	1.71E−13
Anatomical structure development	GO:0048856	1.16E−12
Synapse	GO:0045202	1.51E−11
Aldosterone synthesis and secretion	KEGG:04925	2.54E−03
Cushing syndrome	KEGG:04934	5.01E−03
Cortisol synthesis and secretion	KEGG:04927	9.20E−03
Cholinergic synapse	KEGG:04725	1.00E−02
Oxytocin signaling pathway	KEGG:04921	1.39E−02
Endocrine resistance	KEGG:01522	1.54E−02

Regarding the eating disorders and the specific nutritional trajectory observed in PWS, we explored the connection between PWS DMRs and the genes related to addiction and obesity. The results revealed that 18 of the DMR genes were associated with addiction and obesity (*ADCY3; ADCY9; ATF4; CDK5R1; CHRNB2; GABRD; GABRG3; GNB1; GNB3; GRK5; HDAC4; HDAC9; MAP2K1; PDE11A; PDE2A; PDE3A; PPP1CA; SLC6A3*). The Venn diagram (Fig. 2b) represents this analysis.

### Methylation status

**Fig. 3:**
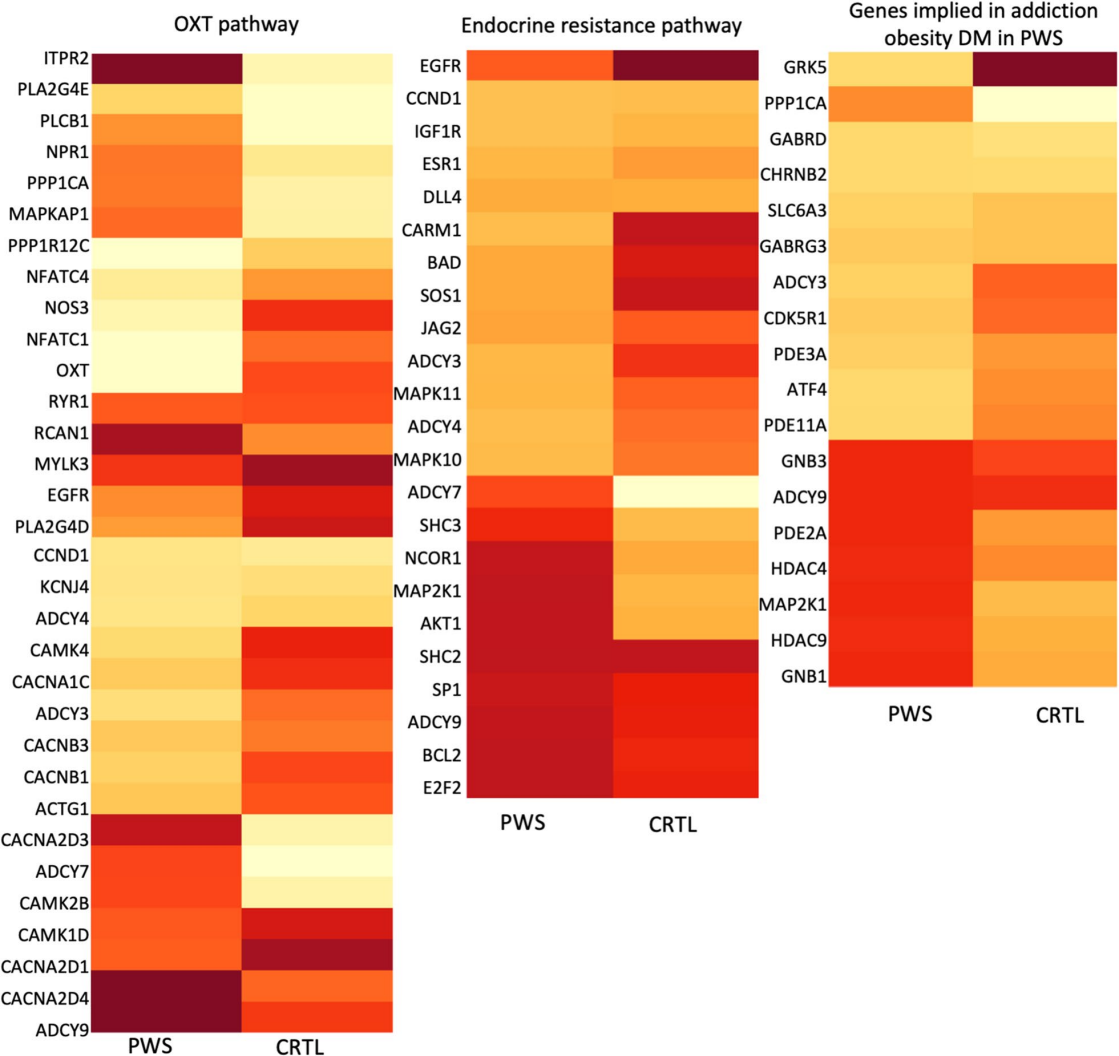
Representation of the methylation level of the gene of interest. The darkness of the color indicates the level of methylation (pale colors indicate low level of methylation, while dark colors indicate high level of methylation)

Twelve of the 32 genes involved in the OXT pathway were hypermethylated in PWS versus control, while the OXT gene was hypomethylated. Ten of the 23 genes involved in the endocrine resistance pathway were hypermethylated in PWS versus control. Eight of the 18 genes involved in obesity and addiction were hypermethylated in PWS versus control. Figure 3 details the methylation level for each gene.

### *SNORD116* and *MAGEL2* mutations

The complete and partial PWS phenotypes were associated, respectively, with *SNORD116* and *MAGEL2* deficits.

The *SNORD116* gene corresponds to the so-called minimal critical region that determines the PWS phenotype, and indeed, patients with *SNORD116* MD display a phenotype very close to that of PWS [18]. The patient with the *SNORD116* MD that we analyzed in this study was the one we previously reported [18]. In order to determine the methylated genes associated with this deletion, we compared the gene methylation in the *SNORD116* patient (a young adult female) with the gene methylation of an infant female control patient. Cellular metabolic processes, nervous system development and metabolic processes were associated with the *SNORD116* MD.

The patient with the *MAGEL2* deletion analyzed in this study was a young adult male. Cellular macromolecule biosynthetic processes, macromolecule biosynthetic processes and organic substance biosynthetic processes were associated with *MAGEL2* inactivation. We compared the gene methylation in the *MAGEL2* patient (a young adult male) with the gene methylation of an infant male control patient.

The top significant results for *MAGEL2* and *SNORD116* are presented in Table 4.

**Table 4:** Top biological processes and KEEG pathways connected to the MAGEL2 mutation and SNORD116 deletion

MAGEL2	Cellular macromolecule biosynthetic process	GO:0034645	2.83E−07
	Macromolecule biosynthetic process	GO:0009059	7.87E−07
	Organic substance biosynthetic process	GO:1901576	3.08E−06
	Biosynthetic process	GO:0009058	4.21E−06
	Cellular biosynthetic process	GO:0044249	9.83E−06
	Regulation of RNA metabolic process	GO:0051252	5.03E−05
	Nucleobase-containing compound metabolic process	GO:0006139	6.72E−05
	Nucleic acid metabolic process	GO:0090304	8.69E−05
SNORD116	Cellular metabolic process	GO:0044237	8.54E−09
	Nervous system development	GO:0007399	9.56E−09
	Metabolic process	GO:0008152	6.57E−08
	Primary metabolic process	GO:0044238	3.31E−07
	Nitrogen compound metabolic process	GO:0006807	5.41E−07
	Nucleic acid metabolic process	GO:0090304	9.97E−07
	Organic substance metabolic process	GO:0071704	1.15E−06
	Central nervous system development	GO:0007417	1.34E−06
	Hippo signaling pathway	KEGG:04390	6.29E−03
	Chronic myeloid leukemia	KEGG:05220	1.25E−02
	Neurotrophin signaling pathway	KEGG:04722	4.77E−02

## Discussion

In this study, we found 5,308 DMRs that matched with 2280 genes in the PWS group and found differences between the analyzed genotypes. These genes are involved in nervous system development, which is relevant to the clinical definition of PWS as a genetic NDD, and are related to the main characteristics and comorbidities of the disease, such as eating disorders with increased fatness and obesity, behavioral disturbances and various comorbidities including endocrine dysfunction, an impaired oxytocin pathway with poor social abilities, addictive behaviors comprising food, smoking and alcohol addiction and dysautonomy. In addition, although poorly documented in the literature, increased sensitivity to drugs has been observed and may be related to these genes [21].

We found DMRs in genes involved in endocrine resistance/sensitivity. Interestingly, patients with PWS display endocrine deficits and are very sensitive to growth hormone (GH) treatment for unknown reasons. We demonstrated in a previous study that children with PWS are more sensitive to GH treatment and need lower doses [22]. Considering our findings, we suggest that this high sensitivity might be partly due to the methylation changes of this pathway. With regard to the endocrine dysfunctions associated with DMRs for the aldosterone pathway, a few cases of unexplained severe hyponatremia were reported in patients with PWS, although the levels of aldosterone and renin were normal [23]. For the cortisol pathway, several reports have hypothesized that PWS patients display central adrenal insufficiency under stressful conditions [24], and small-sized adrenal glands have been documented in autopsies from some cases [25]. However, two recent reports found no cases of central adrenal insufficiency [26, 27], and Ota et al. found that the cortisol response to insulin was normal in patients with PWS, with a peak response that was nevertheless delayed [28].

Notably, we found DMRs in genes involved in the OXT pathway. OXT is a neuropeptide that acts as a neuromodulator in social behavior and a circulating hormone that plays a major role in labor, birth and lactation maintenance. A social deficit has been described in PWS that resembles ASD and can be mild or severe [7]. A dysfunction in the OXT pathway has also been reported, including a hypothalamic OXT prohormone processing deficit and secretion dysfunction [29, 30] and a reduction in the number of hypothalamic OXT neurons [4] with increased plasma levels of OXT [31]. Moreover, we demonstrated in clinical studies that the altered social behaviors and poor sucking are improved in neonates with PWS by OXT treatment administered by the intranasal route [32, 33].

PWS is characterized by a well-described developmental trajectory in terms of nutritional aspects [6, 34]. Indeed, infants with PWS suffer from anorexia in the first so-called nutritional phase [35], with a subsequent shift to hyperphagia with a lack of satiety, leading to early severe obesity [36]. The hyperphagia that most older children and adults display is similar to addictive behavior for food [34]. We found an overlap between PWS DMRs and genes associated with addiction and obesity. Among them, the *ADCY3* gene that encodes for adenylate cyclase 3, which plays an essential role in energy metabolism [37], was hypomethylated in patients with PWS. Interestingly, very recent studies linking novel *ADCY3* variants to obesity and diabetes have been published [37], and *ADCY3* gene mutations with loss of function have been identified in monogenic severe obesity [38]. The melanocortin 4 receptor gene, *MC4R,* is a key component of the melanocortin system, and its mutation is the most common monogenic cause of severe obesity. Interestingly, *MC4R* and *ADCY3* were specifically colocalized in the primary cilia of a subset of hypothalamic paraventricular nucleus neurons [39]. Moreover, specific inhibition of *ADCY3* in the primary cilia resulted in increased food intake and significant weight gain.

We found that *SNORD116* MD DMRs are associated with the Hippo signaling pathway, which is associated with the metabolic processes related to chemical reactions and pathways, including anabolism and catabolism and adaptive thermogenesis. A previous study showed that *Snord116*-deleted mice housed at 22 °C exhibited low body weight, hyperphagia and changes in energy expenditure compared to wild type, and most of these modifications were rescued when the mice were housed at 30 °C [40]. Interestingly, patients with PWS display temperature regulation defects that are most often characterized by episodes of low temperature [40]. Animal models also support a role for the Hippo pathway in regulating adipose cell proliferation, differentiation and adipogenesis [41, 42]. Patients with PWS show unusual body composition and fatness patterns, characterized by reduced lean tissue and increased subcutaneous adiposity [43]. We also found that *SNORD116* MD DMRs are associated with the neurotrophin signaling pathway. A lower *BDNF* level in plasma was found in patients with PWS, as well as lower *BDNF* transcription in human hypothalamus [44, 45]. Last, we found that *SNORD116* MD DMRs were associated with the pathway of chronic myeloid leukemia (CML). One study reported an excess of CML in patients with PWS. In this study of 1160 patients, eight presented leukemia, a prevalence that was 40 times higher than expected. This suggests that the risk of myeloid leukemia may be increased in PWS [46].

Whereas the DMRs associated with *SNORD116* MD were found in genes involved in neurodevelopmental and metabolic pathways, the specific inactivation of *MAGEL2* mutation showed overlap with the genes involved in macromolecule biosynthesis. The truncating point mutations of the paternally inherited allele of *MAGEL2* cause SYS, which has significant phenotypical overlap with PWS, particularly the initial nutritional phase and endocrine dysfunction. However, the developmental trajectory of SYS is clinically distinct, with a particularly high prevalence of ASD (up to 75% of affected individuals) [47], severe intellectual disability and a lower incidence of hyperphagia and obesity.

We acknowledge that this pilot study has several limitations. Notably, we used a small number of samples, especially for the control samples, which carried the risk of high variability between the individuals. The control samples were from infants as two of the PWS samples and this could limit the effects of age and environmental factors. However, the others samples were obtained from children or adults. Moreover, cluster analysis showed that the control patients clustered together and that age did not seem to play a major role in the clustering. Interestingly, the analysis revealed that the patients with DT1*,* UPD or *MAGEL2* mutations were more distant from the controls. The DT1 mutation is associated with more severe clinical symptoms, with these patients scoring lower in adaptive behavior scores and showing poorer reading, math and visual-motor skills [48]. The patient with DT1 (PWS2) clustered apart and displayed not only severe relationship impairment with a need for psychiatric follow-up, but also presented orthopedic problems, with scoliosis and extreme fatigability requiring a wheelchair to move. Patients with UPD more frequently display severe social impairment, including ASD [49]. This suggests that the largest deletions might accentuate the epigenetic modifications observed in the shortest deletions.

Second, our analyses were conducted in blood samples, whereas epigenetic modulation may be tissue- or cell type-specific. Yet, access to specific human tissue, especially brain tissue, is complicated, and most studies focused on DMRs are currently conducted in blood samples.

Third, we chose the analysis of methylation located in the intragenic regions as a first step. However, we acknowledge that methylation occurs in intergenic regions such as the TSS and TE regions and that regions may play a role in epigenetic regulation and phenotype expression.

Last, the DMR analyses accounted for differences in hypermethylation or hypomethylation between patients and controls, therefore suggesting a difference in gene expression. We did not perform RNA sequencing in this study because RNA samples were unavailable. We nevertheless suspect that RNA sequencing would have brought relevant additional information on gene expression.

Despite several limitations, our preliminary results showed that the DMRs we described may be related to the complex phenotype of PWS.

## Conclusion

These data suggest that genetic defects of the imprinted chromosomal region 15q11–q13 that lead to PWS are associated with epigenetic methylation signatures. Those epigenetic signatures are associated with pathways involved in brain development, endocrine function and metabolism. The *SNORD116* MD and *MAGEL2* mutations are also associated with specificities in DMRs that may explain at least partly the complex PWS phenotype. A question of utmost importance arises from these results concerning whether it would be possible to modify the methylation status caused by a lack of expression of *SNORD116*, *MAGEL2* and perhaps other genes in the PWS region [50] with, for example, oxytocin treatment [33] or other drugs and/or social disability rehabilitation.

## Methods

The 15q11–q13 deletions enabled the mapping of three main break sites, BP1 (for breakpoint 1) for the most centromeric, BP2, and BP3 for the most telomeric. These three break sites cause two classes of deletions commonly called type 1 when they extend from BP1 to BP3 and type 2 (the more frequent) when they are between BP2 and BP3 (the type 1 deletion, which is more extensive, therefore includes the type 2 deletion). Uniparental disomy (UPD) refers to the situation in which two copies of the 15q11–q13 region come from the mother.

We collected nine blood samples, seven of which were collected from patients with the PWS phenotype. Two patients with PWS carried a type 2 deletion, two patients a type 1 deletion, one patient a maternal UPD, one patient a microdeletion of a region encompassing *SNORD116*, *IPW* and *SNORD109A* as described by Bieth et al. 2015 [18], and one patient displaying SYS showed a de novo frameshift mutation c.2855delC of the paternal *MAGEL2* gene. For control, we used DNA samples from infants that had been studied for suspicion of genetic diseases that was not confirmed by the genetic analysis and the clinical follow-up. Two blood samples were collected from control patients: a male infant and a female infant.

### Ethics

Before the study for the genetic analysis, in accordance with French law, adult patients gave informed consent if possible or legal guardians in cases of intellectual disabilities, parents gave informed consent for their children. All data were anonymized without any possibility of returning to the patient data. The protocol was submitted to an ethics committee in agreement with the French Jardé law (agreement of the Comité de Protection des Personnes: CPP Sud Ouest et Outremer 1).

### DNA extraction

The DNA was extracted from whole blood, and erythrocytes were lysed by a low salt buffer with Tris–HCl, KCl, MgCl2 and EDTA (TKM1 buffer). The samples were digested by proteinase K and precipitated in sodium acetate*,* then with phenol–chloroform–isoamyl alcohol (PCI), then with chloroform–isoamyl alcohol (CIA) and cold ethanol (99.5%). Last, a DNA pellet was dried in fresh air for 10 min and then suspended in 20 µL of ultra-pure water. DNA concentrations ranged from 70 µg/mL to 100 µg/mL. The samples were conserved in Tris–EDTA pH 8.0 buffer.

Before reduced representation bisulfite sequencing (RRBS) analysis, the 260/230 and 260/280 ratios were measured. In cases of low quality of sample purity, we proceeded to the material purification on AMPure XP beads (Beckman #A63881).

### RRBS analysis

The samples were controlled and validated by microfluorometry with the Qubit High-Sensitivity Assay (Life Technologies #Q32851) and agarose gel 0.8% to monitor degradation. The required quality was 200 ng per sample and a minimal concentration of 4 ng/µl. We did not use any heparin in the sample collection as it interferes with bisulfite labeling. The samples were treated with DNAase-free, protease-free RNAse A (Life Technologies #EN0531).

The banks were constructed by enzymatic digestion of DNA by Mspl enzyme (CCGG) enriched in CpG islands. The digested DNA was repaired in extremities and adenylated in 3’ before being treated with bisulfite and amplified by PCR (13 to 15 cycles depending on the pool). The treatment by bisulfite was followed by a quality check. In our experiment, the rate of non-conversion ranged between 0.4% and 1.24%, indicating that the conversion stage was successful.

The clustering and sequencing steps were performed on a NovaSeq 6000 from Illumina using sequencing-by-synthesis (SBS) technology with NovaSeq Reagent Kits (100 cycles). Clusters were generated by denaturation and dilution of the banks. Then, hybridization and clonal expansion were performed on the flow cell (ID AHHMF2DRXX) using a dual indexing method. The thymine was marked with a green fluorophore and the cytosine with a red fluorophore. Image analysis was performed with NovaSeq Control software and base calling with RTA software, both from Illumina. This step serves to correct the intensity and transform it to a nucleotide base in order to obtain sequences.

The quality of the sequences was controlled with FastQC (v0.11.8). This analysis showed good quality with a probability of error of 2/10,000 (NovaSeq score = 37). Base calling reveals that no cycle was affected by base loss (N bases). The contaminant search was performed with the FastQ Screen software with Bowtie2, and this step revealed no contamination.

### Bioinformatics analysis

The adaptor sequences were then trimmed with Trim Galore!, and the reads were aligned on the genome of reference [51] using Bismark software [52]. The data were extracted from the BAM files with the Bismark methylation extractor tool.

The statistical analysis was performed with the methylSig R package [53] based on a statistical method, with a beta binomial model then applied to calculate the DMRs encompassing a window of 25pb. The sites that were tested only corresponded to the CpG island. The q-value threshold (p-value after correction for the multiple test of Benjamini–Hochberg [54]) was 5%, and the threshold of methylation percentage difference was 25. The DMRs were annotated according to the nearest gene using the RefGene getnearestgene tool from CisGenome [55].

For the individual analysis, we considered as significant a methylation percentage difference of 25 with the control. This method was used for clustering analysis for the *SNORD116* deletion and the *MAGEL2* mutation analysis.

### Hierarchical clustering and pathway analysis

We performed a hierarchical clustering including the gender, the age and the DMRs.

Gene ontology (GO) analysis was conducted with g:Profiler (http://biit.cs.ut.ee/gprofiler/) by following a g:GOSt–functional enrichment analysis. This currently covers KEGG [56], Reactome [57] and WikiPathways [58]; miRNA targets from miRTarBase [59] and regulatory motif matches from TRANSFAC [60]; tissue specificity based on expression data from the Human Protein Atlas [61]; data on protein complexes from CORUM [62]; and human disease phenotype associations from the Human Phenotype Ontology [63].

The list of genes associated with addiction was constituted by the addition of the list of genes collected from KEGG for cocaine addiction (KEGG:05,030), amphetamine addiction (KEGG:05,031), morphine addiction (KEGG:05,032), nicotine addiction (KEGG:05,033) and alcoholism (KEEG:05,034). The genes associated with obesity were extracted from GeneCards® https://www.genecards.org/Search/Keyword?queryString=obesity.

## Data Availability

The datasets used and/or analyzed during the current study are available from the corresponding author on reasonable request.

## Abbreviations

ADHD: Attention deficit hyperactivity disorder
ASD: Autism spectrum disorder
ATP: Adenosine triphosphate
cAMP: Cyclic adenosine monophosphate
CML: Chronic myeloid leukemia
CPP: Comité de Protection des Personnes
DMR: Differentially methylated regions
GABA: Gamma-aminobutyric acid
GO: Gene ontology
GWMA: Genome-wide methylation analysis
IPSC: Induced pluripotent stem cells
MLID: Multilocus imprinting disturbances
NDD: Neurodevelopmental disorder
OXT: Oxytocin
PWS: Prader–Willi syndrome
RRBS: Reduced representation bisulfite sequencing
TE: Transposable elements
TSS: Transcription start sites
